# Mental issues, internet addiction and quality of life predict burnout among Hungarian teachers: a machine learning analysis

**DOI:** 10.1186/s12889-024-19797-9

**Published:** 2024-08-27

**Authors:** Gergely Feher, Krisztian Kapus, Antal Tibold, Zoltan Banko, Gyula Berke, Boroka Gacs, Imre Varadi, Rita Nyulas, Andras Matuz

**Affiliations:** 1 Centre for Occupational Medicine, Medical School, University of Pécs, Pécs, Hungary; 2 Department of Labour Law and Social Security Law, Faculty of Law, University of Pécs, Pécs, Hungary; 3 Department of Behavioural Sciences, Medical School, University of Pécs, Szigeti str. 12, Pécs, 7624 Hungary; 4 Baranya County SZC Zipernowsky Károly Technical School, Pécs, Hungary; 5 Szentágothai Research Centre, University of Pécs, Pécs, Hungary

**Keywords:** Burnout, Teacher, Machine learning, Depression, Insomnia, Quality of life, Internet addiction

## Abstract

**Background:**

Burnout is usually defined as a state of emotional, physical, and mental exhaustion that affects people in various professions (e.g. physicians, nurses, teachers). The consequences of burnout involve decreased motivation, productivity, and overall diminished well-being. The machine learning-based prediction of burnout has therefore become the focus of recent research. In this study, the aim was to detect burnout using machine learning and to identify its most important predictors in a sample of Hungarian high-school teachers.

**Methods:**

The final sample consisted of 1,576 high-school teachers (522 male), who completed a survey including various sociodemographic and health-related questions and psychological questionnaires. Specifically, depression, insomnia, internet habits (e.g. when and why one uses the internet) and problematic internet usage were among the most important predictors tested in this study. Supervised classification algorithms were trained to detect burnout assessed by two well-known burnout questionnaires. Feature selection was conducted using recursive feature elimination. Hyperparameters were tuned via grid search with 10-fold cross-validation. Due to class imbalance, class weights (i.e. cost-sensitive learning), downsampling and a hybrid method (SMOTE-ENN) were applied in separate analyses. The final model evaluation was carried out on a previously unseen holdout test sample.

**Results:**

Burnout was detected in 19.7% of the teachers included in the final dataset. The best predictive performance on the holdout test sample was achieved by random forest with class weigths (AUC = 0.811; balanced accuracy = 0.745, sensitivity = 0.765; specificity = 0.726). The best predictors of burnout were Beck’s Depression Inventory scores, Athen’s Insomnia Scale scores, subscales of the Problematic Internet Use Questionnaire and self-reported current health status.

**Conclusions:**

The performances of the algorithms were comparable with previous studies; however, it is important to note that we tested our models on previously unseen holdout samples suggesting higher levels of generalizability. Another remarkable finding is that besides depression and insomnia, other variables such as problematic internet use and time spent online also turned out to be important predictors of burnout.

**Supplementary Information:**

The online version contains supplementary material available at 10.1186/s12889-024-19797-9.

## Introduction

### The phenomenon and prevalence of burnout

Despite extensive research in the past five decades and its close relationship with mental and physical illnesses, burnout is not labeled as a medical condition but rather considered as an occupational phenomenon [1]. In line with this, a large body of research focused on workplace factors and revealed that they play an important role in the development of burnout [2]. However, individual-level factors such as mental health-related factors or psychological traits also contribute to burnout symptoms [3]. Burnout was initially considered to develop among those working in helping professions, but recent studies showed similar prevalence rates among “blue-collar” workers, while other forms, for example, parental burnout can also develop [4–6]. The overall prevalence of burnout can vary between 12 and 60% depending on the target population, probably reaching its peak among healthcare workers, but the results may be influenced by the fact that studies including medical/nursing students and healthcare workers are far overrepresented [3, 6–8].

### Causes of burnout and associated phenomena

The most important work-related risk factors of burnout are high demands, high workload, low job control, low reward, workplace injustice and job insecurity based on a relatively recent meta-analysis [2]. While the importance of work-related factors is high, the effects of mental health related factors and psychological traits such as neuroticism and emotional labor cannot be neglected. In fact, their negative effects might be comparable to the role of workplace stressors [4, 7]. In addition, sociodemographic factors have also been associated with burnout: to name a few, younger age, living alone, being single, and having no or little workplace experience are also important risk factors of the burnout [4–6, 8].

It is generally agreed and supported by cumulative evidence that burnout is the result of a long-standing procedure provoked by prolonged emotional strain and stress leading to behavioural and self-esteem disorders, with markedly impaired coping strategies [7]. To cope with the increased stress, affected individuals tend to turn to addictive behaviours such as extensive smoking, heavy drinking or problematic usage of the internet (see e.g. 9,10). Compared to “conventional” addictions, internet addiction (IA) or problematic usage of the internet (PUI) is a relatively new term [9, 10]. Despite extensive research on the topic, IA is still labelled as a phenomenon and not as a medical condition, however, it seems to be associated with several mental and physical conditions including burnout [9]There is also a strong association between burnout and physical as well as mental illnesses such as insomnia, depression, hospitalization for mental disorders, cardiovascular syndromes and mortality [11]. Both depression and burnout have relatively similar symptomatology such as anhedonia, insomnia, loss of social functions, feeling of worthlessness etc. raising the possibility of the same phenomenon. However, a recent meta-analysis showed that there is no conclusive overlap between depression and burnout suggesting that they might be different constructs [12].

Insomnia is a frequent disease that affects 22% of the whole population with female predominance [13]. It can occur as an independent disease, but it is often intertwined with mental illnesses, such as depression or anxiety. Furthermore, the greater presence of insomnia is associated with higher level of burnout based on a recent meta-analysis [14].

Quality of life (QOL) is a relatively new term suggested to replace the words “happiness” or “well-being”, with the aim of covering all aspects of life. QOL means the individual’s impression of their life situation taking values, aims, worries and prospects into account as defined by the WHO [15]. Based on the above-mentioned results, it is not surprising that the development and severity of burnout negatively affect the individual’s quality of life [15], highlighting the importance of research investigating the etiology of burnout.

### Research on burnout in teachers

Similar to healthcare workers, the prevalence of burnout in teachers is also relatively high: on average, the prevalence might be around 15% [16] but it can as high as 53% [17]. The presence of burnout in teachers might be explained by the fact that teaching is a psychologically demanding job with high levels of stress [18]. Given the importance of the topic, many studies investigated the potential predictors of burnout in teachers. A recent meta-analysis aiming to reveal the association between burnout and Big Five personality dimensions found that all personality dimension expect from neuroticism were negatively related to the severity of burnout, however, the effect sizes were small to moderate [19]. This suggests that factors other than personality also have to explain individual differences in teacher’s burnout. In line with this, a systematic review of longitudinal studies identified the key predictors of burnout as job satisfaction, work climate (including pressure) and teacher self-efficacy [20]. It has also been pointed out that the effects of school climate involving teacher-student relations, administration etc. might be mediated by the level of satisfaction [21].

### Limitations of prior research and the need for machine learning

Most previous studies used “conventional” statistical methods for the prediction of burnout syndrome measured by a single questionnaire, for example, the Maslach’s Burnout Inventory (MBI), the Copenhagen Burnout Inventory (CBI) or the Oldenburg Burnout Inventory (OLBI) [see e.g. 22–24]. However, as burnout is a complex phenomenon that is affected by many different factors, the use of machine learning (ML) for its prediction would be beneficial because ML algorithms are capable of handling large, complex datasets and they can potentially identify the best predictors of burnout. In fact, the use of ML is advantageous as it is able to detect non-linear associations and complex interactions. For example, the non-linear methods, decision trees (DT) and random forests (RF), are typically used when the aim is to unravel interactions between variables [25, 26]. Another asset of using RF is that they usually show high levels of predictive accuracy [27]. Similarly, support vector machines (SVM) are also among the most frequently used ML algorithms capable of detecting non-linear associations in the data [28]. The predictive accuracy of SVMs are also comparable with RF and SVMs often even outperform RFs in medical research [29, 30].

In line with the advantages of using ML, a few recent studies applied ML methods to detect the phenomenon in various samples, for instance, in surgery trainees [31], nurses [32], front-line workers [33], healthcare professionals [34], start-up directors and representatives [35], and teachers [36]. Although these studies are important pioneer studies in the ML-based prediction of burnout, a general criticism of them is that they all used only one questionnaire to assess burnout. This in turn makes the models more prone to the potentially compromised psychometric properties of the instruments. In addition, as can be seen from the list above, most of the ML analyses were conducted on a sample of healthcare workers, and – to the best of our knowledge – only a few studies have so far focused on burnout prediction in teachers [36–38].

The studies applying ML for the prediction of burnout investigated various predictor variables. A study conducted during the COVID-19 pandemic tested contextual factors associated with teaching as well as personal factors, for example, basic job characteristics including institutional characteristics, professional development, social support, current personal concerns etc. [36]. Another study that was also carried out during the pandemic, specifically targeted variables associated with the stress induced by COVID-19 and the way how teachers coped with the situation [39]. According to these studies, the best predictors of burnout included coping strategies, personal concerns about one’s own and loved ones’ health, information overload etc. In addition, the importance of income, overtime working, frequent headaches were also emphasized in a sample of Columbian school teachers [38]. Despite including similar samples in terms of occupation, these studies are rather heterogeneous as they worked with vastly different sample sizes (ranging from 54 to 936), features and algorithms. In line with this, the reported predictive accuracies also varied strongly (ranging between approx. 70% and 97%) [36, 38, 39]. Therefore, it would be important to gain more information on the ML-based prediction of burnout in teachers.

### Study aims

All in all, teachers might potentially be an important target population of ML studies aiming to predict burnout. Teaching is a demanding job, and teachers have to face significant challenges, especially in the 21st century (e.g. due to globalization and digitalization) and have to adapt their roles in the education of students [40]. Apart from teaching, their work is burdened with administrative tasks as well as personal conflicts with students, colleagues and parents, so teachers are among the most vulnerable in the development of burnout [41]. Therefore, in this study, we used a dataset collected from high school teachers to train ML algorithms for the detection of burnout. As there is no standard questionnaire-based method for the detection of the presence of burnout, we administered two widely-used questionnaires, and teachers were categorized into the “burnout group” only if they met the criteria of both questionnaires. In addition, the other main aim of this study was to identify the psychological (e.g. depression, insomnia, internet addiction etc.) and sociodemographic (e.g. gender, age, internet using habits etc.) predictors of burnout.

## Materials and methods

### Participants

This cross-sectional, paper-based questionnaire study was carried out between January 2020 and August 2020. The study recruited high-school teachers in 14 large educational sites in Middle and East Hungary (the Acknowledgement part contains their names). Based on the collected data we have already published two articles; however, it is important to note that those studies had completely different aims and analyses [42, 43]. The sample used for the current analysis contained data from 1,665 high-school teachers (565 male, 1,100 female). The study protocol, documentation and the used questionnaire were approved by the Ethics Committee of the University of Pecs (license number 8434-PTE 2020). Informed consent was read and signed by participants prior to delivery.

### Instruments

#### Sociodemographic, medical, and internet usage related questions

Included demographic data, risk factors and medical conditions were age, gender, marital status, number of children, type of work, years spent with work, work schedule, tobacco use, alcohol and illicit drug use, the presence of diabetes, hypertension, ischemic heart disease, history of musculoskeletal pain and depression. Participants were also asked to rate the subjective level of their current health status (CHS) on a 100-point scale. Goals of being online (e.g. gaming, social media, work etc.), daily time spent online and time intervals (i.e. 3-hour intervals during the whole course of the day starting from 12 a.m.) were also collected. For a full list of questions, please, see Supplementary Table S1.

#### Problematic internet use questionnaire

Problematic usage of the internet was detected by the Problematic Internet Use Questionnaire (PIUQ) developed by Demetrovics et al. as we have previously published [44]. This 18-item questionnaire contains three main subscales: obsession, neglect and control disorder. The questionnaire contains 18 items, which can be divided into three main parts namely obsession, neglect and control disorder. Obsession subscale refers to obsessive thinking about the Internet (daydreaming, rumination, and fantasizing) and withdrawal symptoms caused by the lack of Internet use (anxiety and depression) (“*How often do you feel tense*, *irritated*, *or stressed if you cannot use the Internet for as long as you want to?*”). Neglect subscale contains items about neglecting everyday activities, social life, and essential needs (“*How often do you spend time online when you’d rather sleep?*”). Control disorder subscale reflects difficulties in controlling time spent on the Internet (“*How often do you realize saying when you are online*, *“just a couple of more minutes and I will stop?*”). Each of them consists of six questions. The answers are scored on a 5-point Likert-type scale ranging from 1 (never) to 5 (always). A total score exceeding 41 points suggests Internet addiction [44]. Reliability of each questionnaire subscale was assessed using McDonalds’s ω [following 45]. In our sample, all three subscales of PIUQ showed adequate reliability (Control disorder: ω = .79; Obsession: ω = .88; Neglect: ω = .81).

#### Beck’s depression inventory

Depression was detected the short version of Beck Depression Inventory (BDI-SF) [46], which examines the severity of depression using 9 questions and demonstrated good internal consistency in Hungarian samples [47]. The questionnaire assesses the following symptoms: social withdrawal, indecision, sleep disturbance, fatigue, excessive anxiety about physical symptoms, incapacity for work, pessimism, dissatisfaction, lack of joy, self-blame. An example item is “*I have lost all of my interest in other people*”. Each item is rated on a Likert scale ranging from 1 to 4 points. After summarizing the results, we can distinguish between severe (≥ 26 points), moderate (19–25 points), mild depression (10–18 points), or the absence of mood disorder (0–9 points) [47]. The McDonald’s ω value of BDI in our sample was 0.87.

#### Athens insomnia scale

Sleep disturbance was measured with Athens Insomnia Scale (AIS). This questionnaire contains eight items about nocturnal symptoms (difficulty of falling asleep, early awakening), and three items about daytime consequences. The items need to be rated on a scale from 0 to 3. The higher the score, the worse the quality of sleep (maximum 24 points). Having > 6 points suggests the presence of insomnia, while > 10 points indicates clinically significant sleep disturbance (severe insomnia) [48, 49]. The internal consistency of the AIS was excellent McDonald’s ω = 0.90.

#### EuroQol 5 dimensions

The five-dimension EQ-5D (health-related quality of life) questionnaire was applied to assess the quality of life in self-sufficiency (ranging from ‘*I have no problems with self-care*’ to ‘*I am unable to wash or dress myself’*), usual activities (ranging from ‘*I have no problems doing my usual activities*’ to ‘*I am unable to do my usual activities*’), mobility (ranging from ‘*I have no problems walking about’* to ‘*I am confined to bed*’), anxiety/depression (ranging from ‘*I am not anxious or depressed*’ to ‘*I am extremely anxious or depressed*’) and pain/malaise (ranging from ‘*I have no pain or discomfort*’ to ‘*I have extreme pain or discomfort*’) [50]. Items are required to be rated on a 5-points Likert scale.

#### Burnout assessment

Burnout was measured with two different questionnaires. The Maslach Burnout Inventory (MBI) [51, 52], which is generally considered to be the “gold standard” of measurement. has three subscales according to the most widely accepted theory of burnout and these are emotional exhaustion (EE, being overburdened and depleted of resources), depersonalization (DP, distant attitude towards one’s work and/or people) and personal accomplishment (PA, satisfaction with past and present accomplishments). The items refer to the burdensome feeling of education within the last 3 months, for example: “*I feel exhausted by the end of a day spent at work*.” The items are required to be rated on a 7-points Likert scale from 0 (meaning ‘never’) to 6 meaning (‘every day’). High scores on EE and DP, while low scores on PA are indicative of burnout. The overall burnout can be defined as EE score ≥ 27 and/or DP score ≥ 10. Reliability measures for each subscale were sufficiently large: EE (ω = 0.90), DP (ω = 0.78), PA (ω = 0.86).

For the assessment of burnout, we also used the Mini Oldenburg Burnout Inventory (MOLBI) which has shown to have robust psychometric properties in the measurement of occupational burnout [53, 54]. The advantage of this questionnaire includes that it can be used as a universal measurement tool for any profession, and it was specifically designed to reduce the content and theoretical criticisms of the MBI questionnaire. Thus, the statements are specific, and their number is evenly distributed within each scale, with an even distribution of positive–negative items. The questionnaire measures burnout along two dimensions: Exhaustion measures work-related fatigue and the emotional, cognitive and physical strain of work, while the Disappointment subscale measures loss of interest in work, depersonalization, loss of commitment and possible cynicism. Example items: ‘*After work*, *I feel worn out*’ (Exhaustion), ‘*I think less and execute tasks mechanically’* (Disappointment). Half of the items are reversed and respondents are asked to rate the statements a 4-point Likert scale Mean scores ≥ 2.25 on exhaustion and ≥ 2.1 on disappointment are suggested to be used as cutoff values of burnout detection. McDonald’s ω values of Exhaustion and Disappointment were 0.78 and 0.73, respectively.

### Data analysis

All programming was implemented in Python using the scikit-learn (Version 1.0.2.) package [32]. The data set was split into training (∼ 70%) and test sets (∼ 30%). Feature selection via recursive feature elimination (base estimator was a random forest model) with 10-fold cross-validation (10-CV) was performed on the training set. Supervised classification algorithms were used to develop models that are able to detect burnout in teachers. The burnout label was assigned to the data of participants if scores on both burnout questionnaires (i.e. MBI and MOLBI) indicated the presence of burnout (see above). Three supervised classification algorithms were used for burnout detection: support vector machine (SVM), decision tree (DT) and random forest (RF). Hyperparameters for each algorithm were optimized through grid search with 10-CV. For SVM, the hyperparameters C and gamma were tuned with either linear, radial basis function or polynomial kernel. For DT and RF, maximum depth was tuned. In addition, for RF, the number of estimators was also tuned. For training as well as to determine the model performance on the test set, the area under the receiver operating characteristic curve (area under the curve [AUC]) was used. AUC is a measure of the model’s capability of distinguishing between classes independent from classification thresholds. AUC scores range between 0 and 1 with higher scores indicating better classification performance and have recently suggested to be used when the dataset is imbalanced [55]. Moreover, sensitivity, specificity and balanced accuracy were also calculated to evaluate the performance of the test data set. Sensitivity refers to the classifier’s capability of identifying the positive class (i.e. burnout), while specificity assess how successfully the classifier identifies the negative class (i.e. non-burnout). Balanced accuracy refers to the mean of sensitivity and specificity and provides an estimate on how accurate the classifier is in general. Finally, to gain a deeper insight into model performances, we also predicted burnout using logistic regression as a baseline model for comparison with ML models.

The distribution of target labels (i.e. burnout vs. non-burnout) indicated class imbalance. More specifically, for ∼ 19.7% of the participants, the questionnaires indicated the presence of burnout, while the remaining ∼ 80.3% were found not to be burnt out. To deal with the class imbalance, three methods were used, separately. The first method included the training of weighted algorithms (i.e. cost-sensitive learning) [56]. That is, for each algorithm, we set the class weights 4 and 1 for the data labelled “burnout” and “non-burnout”, respectively. The other two methods were applied using the imbalanced-learn package: was as a downsampling method, we applied the Repeated Edited Nearest Neighbours (RENN) algorithm (following [57]), whilst we also applied a hybrid method, the Synthetic Minority Over-sampling Technique with Edited Nearest Neighbours (SMOTE-ENN) [58]. To avoid information leakage, in case of both methods, resampling was carried out in the training set only, within cross-validation [59–61]. That is, the training set was first split into *k* folds and resampling was applied to only *k*-1 folds used for training.

Finally, the whole procedure (i.e., data split, feature selection, model training and model evaluation) was repeated 20 times [62, 63]. Evaluation metrics were computed for each iteration and means as well as the 95% confidence intervals (95% CI) were calculated. In addition, to help the interpretability of the results, the most average iterations (i.e., where the predictive performance of the model was closest to the mean predictive performance) were also selected and the performance of those models are also presented below. Decision trees were also plotted for the most average iteration.

## Results

### Descriptive statistics of the whole sample

The initial dataset consisted of the data of 1,665 high-school teachers. Due to missing information (i.e. missing data on more than 5% of the variables), the data of 89 participants (43 male and 46 female) had to be dropped and thus, the final dataset included 1,576 teachers (522 male and 1,054 female). Characteristics of the final sample are summarized in Table 1.

**Table 1:** Demographic characteristics of the participants in the whole sample (*n* = 1576)

Demographic characteristics / Groups	Burnout (*n* = 311)	Not burnout (*n* = 1265)
	*n* (%)	*n* (%)
Gender		
Male	101 (6.4)	421 (26.7)
Female	210 (13.3)	844 (53.6)
Age group		
18–25 years	5 (0.3)	23 (1.5)
26–35 years	44 (2.8)	155 (9.8)
35–45 years	113 (7.2)	410 (26.0)
46–55 years	86 (5.5)	435 (27.6)
56–62 years	51 (3.2)	184 (11.7)
>62 years	12 (0.8)	56 (3.7)
Work schedule		
Full-time	272 (17.3)	1104 (70.1)
Part-time	39 (2.5)	161 (10.2)

Note: Percentages represent the proportion of each category within the entire sample

### Feature selection

**Fig. 1:**
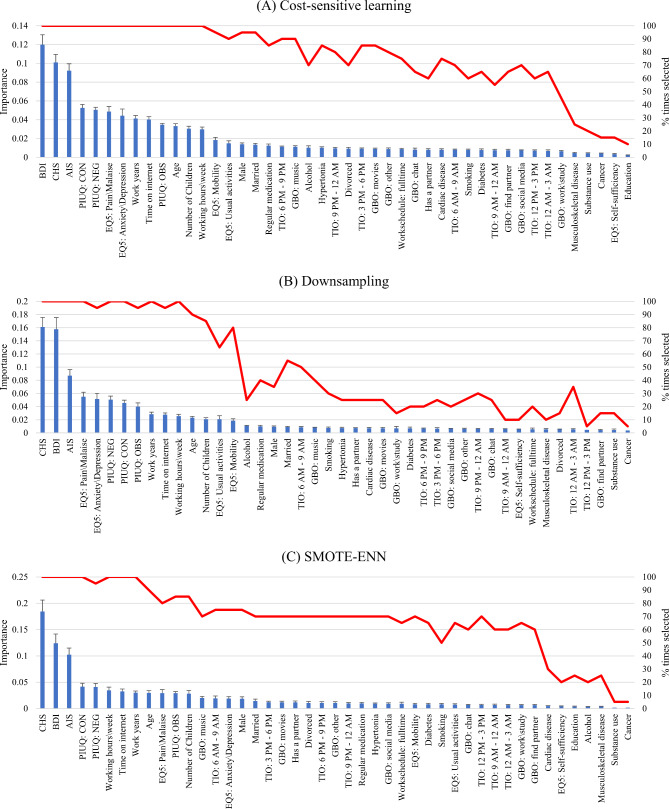
Results of feature selection based on recursive feature elimination carried out (**A**) prior to algorithm training with class weights and (**B**) after downsampling. Error bars represent the standard deviation of importance across twenty iterations. Abbreviations: AIS = Athens Insomnia Scale; BDI = Beck’s Depression Inventory; CHS = Current health status; EQ-5D = EuroQol 5-dimension; GBO = Goals of being online (dummy coded); PIUQ = Problematic Internet Use Questionnaire; TIO = Time interval online (dummy coded)

Descriptive statistics of the most frequently selected features calculated for the whole sample are shown in Table 2. The results of feature selection via recursive feature elimination are presented in Fig. 1. Here we only highlight the most important features. Regardless of which method was used to deal with the imbalanced data, the best three features were BDI, CHS and AIS. The PIUQ subscales Control and Neglect were also among the top predictors as well as the EQ-5D factors, Anxiety\Depression and Pain\Malaise. Other internet-related variables such as the Obsession subscale of PIUQ and the daily time spent with the internet were also among the top 15 features in all three cases. Similarly, two work-related variables, weekly working hours and the number of working years were also among the best features. Other relatively important features included age, sex, family status, the number of children and the Usual activities and Mobility factors of EQ-5D. Feature selection as well as model performances obtained when classifying MOLBI and MBI-based burnout categories separately can be found in the Supplementary materials (see supplementary tables S2 and S3 and figures S1 and S2).

**Table 2:** Descriptive statistics of the top 25 features calculated for the whole sample (*n* = 1576)

Features / Groups	Not burnout (*n* = 1265)	Burnout (*n* = 311)
	Mean (SD) or *n* (%)	Mean (SD) or *n* (%)
*Numerical features*		
Age	3.613 (1.076)	3.547 (1.091)
AIS	3.222 (3.911)	5.540 (4.227)
BDI	10.740 (2.437)	14.193 (4.837)
CHS	85.695 (12.110)	74.39 (18.070)
Daily time spent on the internet	2.013 (1.494)	2.257 (1.636)
Equation 5: Anxiety\Depression	0.130 (0.389)	0.576 (0.745)
Equation 5: Pain\Malaise	0.255 (0.499)	0.704 (0.805)
Equation 5: Usual activities	0.093 (0.350)	0.383 (0.761)
Number of Children	1.477 (1.017)	1.424 (1.000)
PIUQ: Control	8.443 (2.773)	9.865 (3.772)
PIUQ: Neglect	8.146 (2.619)	9.875 (4.237)
PIUQ: Obsession	7.123 (2.095)	8.547 (4.232)
Work years	4.126 (1.346)	4.296 (1.251)
Working hours\week	37.649 (8.151)	38.879 (7.790)
*Categorical features (n and % of „yes”)*		
Alcohol	43 (3.399)	36 (11.576)
Diabetes	92 (7.273)	23 (7.395)
Divorced	152 (12.016)	34 (10.932)
Goal of being online: music	659 (52.095)	164 (52.733)
Hypertonia	270 (21.344)	83 (26.688)
Male	421 (33.281)	101 (32.476)
Married	764 (60.395)	178 (57.235)
Regular medication	307 (24.269)	97 (31.190)
Substance use	20 (1.581)	21 (6.752)
TIO: 9 PM – 12 AM	248 (19.605)	70 (22.508)
TIO: 6 AM – 9 AM	151 (11.937)	52 (16.720)

Abbreviations: AIS = Athens Insomnia Scale; BDI = Beck’s Depression Inventory; CHS = Current Health Status; EQ-5D = EuroQol 5-dimension; n = sample size; PIUQ = Problematic Internet Use Questionnaire; SD = standard deviation; TIO: time interval online

Note: Please, note the Age and Work years were ordinal variables with six and seven categories, respectively

**Fig. 2:**
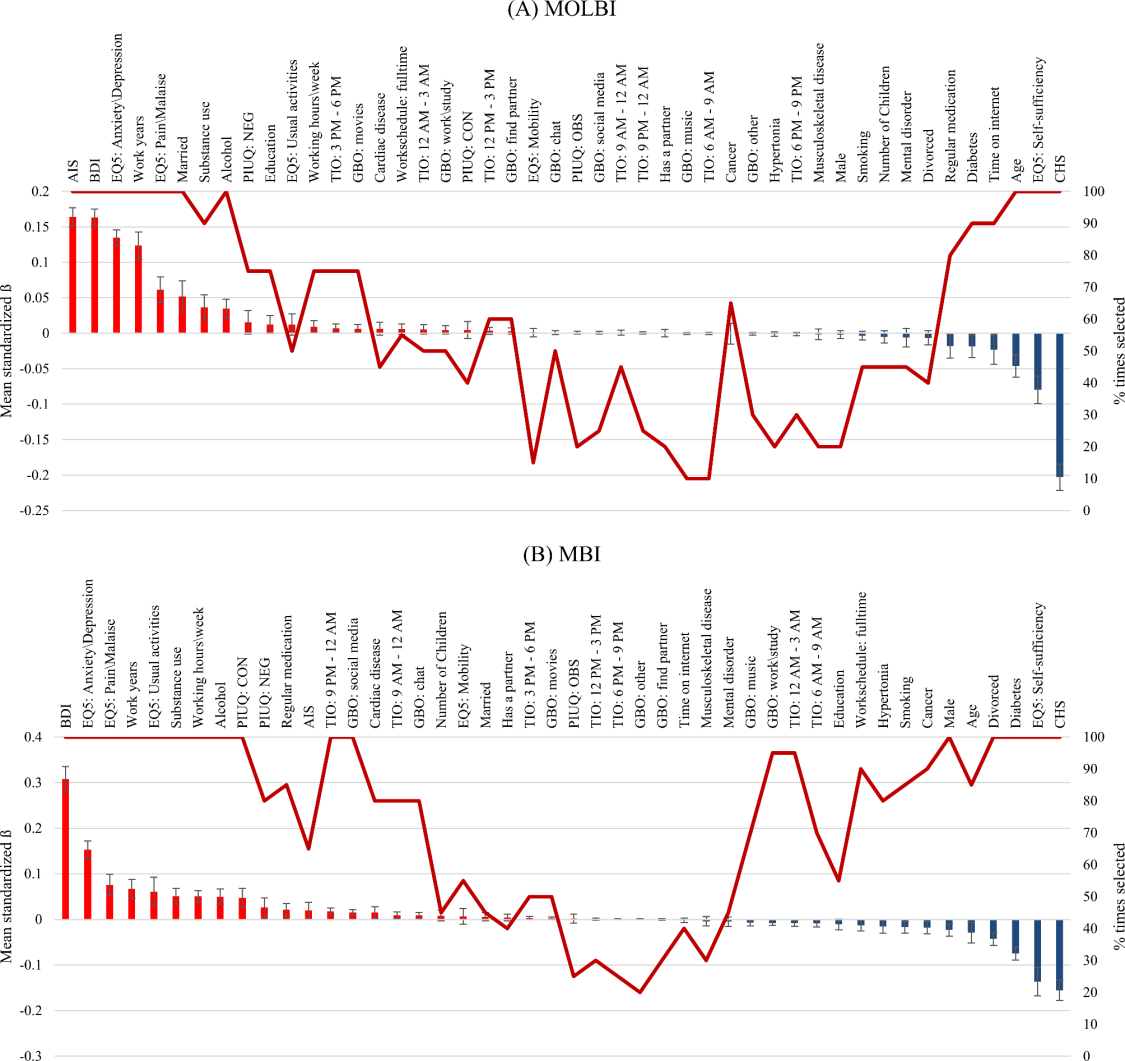
Results of feature selection using elastic-net regression predicting (**A**) Mini Oldenburg Burnout Inventory (MOLBI) total scores and (**B**) Maslach Burnout Inventory (MBI) total scores. Error bars represent the standard deviation across twenty iterations. Positive predictors are indicated by red colour, while negative predictors are indicated by blue colour. Abbreviations: AIS = Athens Insomnia Scale; BDI = Beck’s Depression Inventory; CHS = Current health status; EQ-5D = EuroQol 5-dimension; GBO = Goals of being online (dummy coded); PIUQ = Problematic Internet Use Questionnaire; TIO = Time interval online (dummy coded)

To gain further insight about the association between the features and burnout, elastic net regression with 10-fold CV were also used to predict burnout. However, for the regression analyses, the MOLBI and MBI sum scores were used as outcome variables instead of burnout category. The results of feature selection via elastic net regression are presented in Fig. 2. Here below, we only report the five best positive and negative predictors. The analysis of MOLBI scores revealed positive associations with AIS, BDI, EQ-5D (Pain\Malaise, Anxiety\Depression), and the number of working years, while negative associations were found with CHS, EQ-5D Self-sufficiency, age, daily time spent on the internet and the diagnosis of diabetes. The analysis of MBI showed that the scores were positively associated with BDI, EQ-5D (Anxiety\Depression, Pain\Malaise, Usual activities) and the working years, while negatively associated with CHS, EQ-5D Self-sufficiency, being diagnosed with diabetes, being divorced and age.

### Burnout prediction

Model performances on the test set are summarized in Table 3. Below we only report the most important findings. In the case of cost sensitive learning, the baseline model was only outperformed by RF (i.e. higher AUC, balanced accuracy and sensitivity but lower specificity on the test set). In the case of dowsampling, RF outperformed the baseline model in all evaluation metrics except from sensitivity. DT outperformed the baseline model in balanced accuracy and specificity, while SVM performed better than the baseline model in terms of AUC and specificity. When SMOTE-ENN was used, the SVM performed better than the baseline model in terms of AUC and sensitivity. Similarly, RF also performed better than the baseline model in sensitivity. When tested on the holdout test set, the overall best predictive performance in terms of AUC (0.811) and balanced accuracy (0.745) was achieved when RF was trained with class weights. However, in terms of sensitivity (0.803) the RF algorithm combined with SMOTE-ENN, while in terms of specificity (0.859) SVM combined with downsampling showed the highest performances.

**Table 3:** Results of classification algorithms predicting burnout

Algorithm	Dataset	Evaluation metrics			
		Balanced accuracy (95% CI)	AUC (95% CI)	Sensitivity (95% CI)	Specificity (95% CI)
*Cost-sensitive*					
Baseline model	Training	0.767 (0.761 − 0.773)	0.843 (0.838 − 0.848)	0.728 (0.718 − 0.738)	0.805 (0.801 − 0.809)
	Test	0.722 (0.714 − 0.730)	0.792 (0.785 − 0.799)	0.652 (0.636 − 0.668)	0.792 (0.783 − 0.801)
Decision tree	Training	0.768 (0.761 − 0.775)	0.822 (0.815 − 0.829)	0.766 (0.750 − 0.782)	0.769 (0.753 − 0.785)
	Test	0.735 (0.723 − 0.747)	0.771 (0.755 − 0.787)	0.711 (0.687 − 0.735)	0.759 (0.743 − 0.775)
Random forest	Training	0.817 (0.806 − 0.828)	0.904 (0.892 − 0.916)	0.871 (0.858 − 0.884)	0.763 (0.751 − 0.775)
	Test	0.745 (0.736 − 0.754)	0.811 (0.801 − 0.821)	0.765 (0.750 − 0.780)	0.726 (0.716 − 0.736)
Support vector machine	Training	0.768 (0.742 − 0.794)	0.870 (0.855 − 0.885)	0.678 (0.598 − 0.758)	0.858 (0.823 − 0.893)
	Test	0.692 (0.668 − 0.716)	0.790 (0.782 − 0.798)	0.553 (0.473 − 0.633)	0.831 (0.796 − 0.866)
*Downsampling*					
Baseline model	Training	0.812 (0.804 − 0.820)	0.910 (0.903 − 0.917)	0.685 (0.670 − 0.700)	0.940 (0.935 − 0.945)
	Test	0.713 (0.704 − 0.722)	0.790 (0.779 − 0.801)	0.635 (0.615 − 0.655)	0.791 (0.778 − 0.804)
Decision tree	Training	0.751 (0.744 − 0.758)	0.802 (0.795 − 0.809)	0.724 (0.701 − 0.747)	0.778 (0.760 − 0.796)
	Test	0.717 (0.702 − 0.732)	0.758 (0.745 − 0.771)	0.667 (0.632 − 0.702)	0.767 (0.748 − 0.786)
Random forest	Training	0.768 (0.757 − 0.779)	0.851 (0.840 − 0.862)	0.713 (0.687 − 0.739)	0.823 (0.807 − 0.839)
	Test	0.717 (0.706 − 0.728)	0.797 (0.785 − 0.809)	0.626 (0.602 − 0.650)	0.809 (0.788 − 0.830)
Support vector machine	Training	0.719 (0.691 − 0.747)	0.847 (0.839 − 0.855)	0.566 (0.479 − 0.653)	0.872 (0.839 − 0.905)
	Test	0.679 (0.655 − 0.703)	0.794 (0.784 − 0.804)	0.499 (0.420 − 0.578)	0.859 (0.825 − 0.893)
*SMOTE-ENN*					
Baseline model	Training	0.842 (0.833 − 0.851)	0.923 (0.916 − 0.930)	0.883 (0.871 − 0.895)	0.802 (0.792 − 0.812)
	Test	0.712 (0.701 − 0.723)	0.783 (0.772 − 0.794)	0.780 (0.760 − 0.800)	0.643 (0.629 − 0.657)
Decision tree	Training	0.733 (0.724 − 0.742)	0.790 (0.782 − 0.798)	0.839 (0.820 − 0.858)	0.628 (0.597 − 0.659)
	Test	0.686 (0.670 − 0.702)	0.725 (0.710 − 0.740)	0.761 (0.734 − 0.788)	0.611 (0.582 − 0.640)
Random forest	Training	0.752 (0.741 − 0.763)	0.854 (0.842 − 0.866)	0.880 (0.868 − 0.892)	0.623 (0.601 − 0.645)
	Test	0.698 (0.686 − 0.710)	0.773 (0.760 − 0.786)	0.803 (0.778 − 0.828)	0.594 (0.571 − 0.617)
Support vector machine	Training	0.753 (0.744 − 0.762)	0.828 (0.821 − 0.835)	0.853 (0.835 − 0.871)	0.652 (0.631 − 0.673)
	Test	0.712 (0.700 − 0.724)	0.786 (0.775 − 0.797)	0.789 (0.763 − 0.815)	0.636 (0.611 − 0.661)

Note: AUC = area under the receiver operating characteristic curve; CI = confidence interval; SMOTE-ENN = Synthetic Minority Over-sampling Technique with Edited Nearest Neighbours

**Fig. 3:**
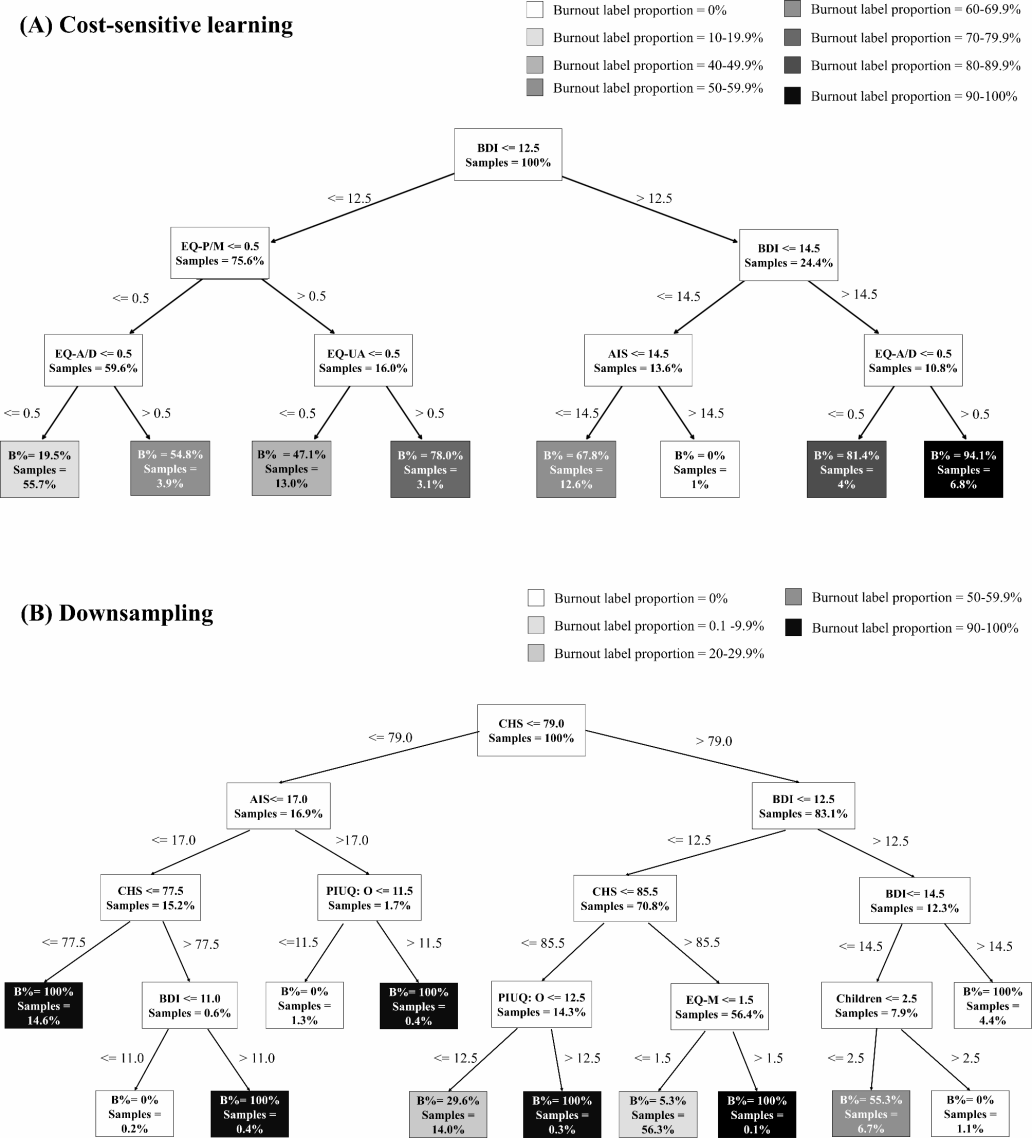
Decision trees trained (**A**) with class weights and (**B**) after downsampling. Abbreviations: B% = proportion of burnout labels in percentages; AIS = Athens Insomnia Scale; BDI = Beck’s Depression Inventory; Children = number of children, CHS = Current health status; EQ-A/D = Anxiety and depression; EQ-M = Mobility; EQ-P/M = Pain and malaise; EQ-UA = Usual activities; PIUQ: O = Obsession subscale of Problematic Internet Use questionnaire

**Fig. 4:**
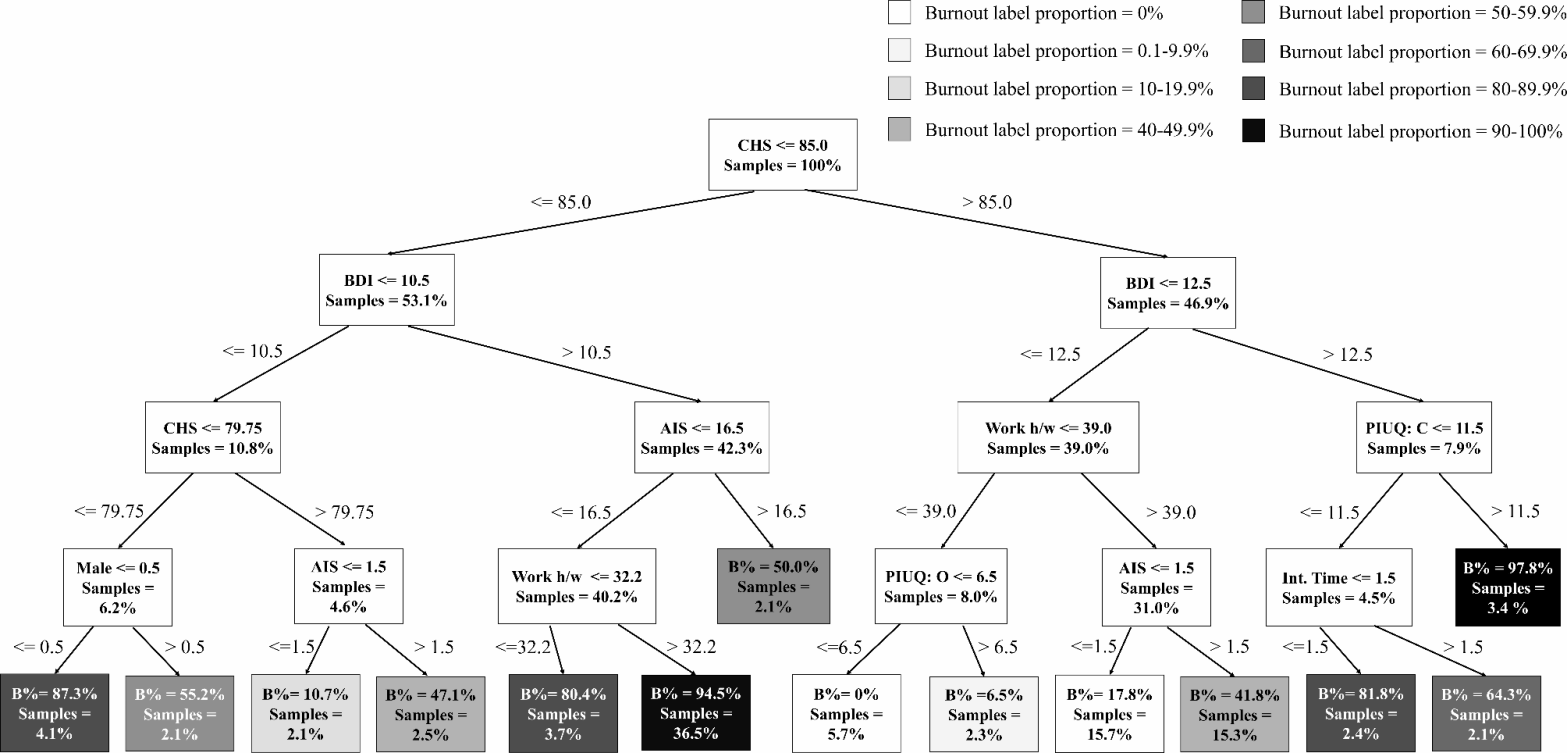
Decision tree combined with SMOTE-ENN. Abbreviations: B% = proportion of burnout labels in percentages; AIS = Athens Insomnia Scale; BDI = Beck’s Depression Inventory; CHS = Current health status; EQ-A/D = Anxiety and depression; Int. time = daily time spent on the internet; PIUQ: C = Control subscale of Problematic Internet Use questionnaire; PIUQ: O = Obsession subscale of Problematic Internet Use questionnaire; Work h/w = weekly working hours

To get more insight into the functioning of the models, the most representative DTs were also visualized (see Figs. 3 and 4). When class weights were applied, the DT (AUC = 0.771; balanced accuracy = 0.707; sensitivity = 0.634; specificity = 0.779 in the test set) closest to the mean model performance used the variables Anxiety\Depression (EQ-5D), Pain\Malaise (EQ-5D), Usual activities (EQ-5D), BDI and AIS (Fig. 3a). When downsampling was applied, the DT (AUC = 0.755; balanced accuracy = 0.732; sensitivity = 0.710; specificity = 0.755 in the test set) closest to the mean model performance used the variables BDI, AIS, CHS, Obsession (PIUQ), Mobility (EQ-5D), and the number of children (Fig. 3b). The most average DT obtained when SMOTE-ENN was applied turned out to be very complex and therefore, hard to interpret. To make it visually comprehensive, we set the minimum number of samples at a leaf node to be 2%. The resulting decision tree (see Fig. 4) had an AUC score of 0.740, balanced accuracy of 0.728, sensitivity of 0.763 and specificity of 0.692.

The elastic net regression models predicting MOLBI scores in the training set, had a mean R^2^ of 0.342 (95% CI = 0.336 − 0.347) with a mean root mean squared error (RMSE) of 0.811(95% CI = 0.808 − 0.815). In the test set, the mean R^2^ was 0.320 (95% CI = 0.306 − 0.333) with a mean RMSE of 0.825 (95% CI = 0.817 − 0.833). The regression analyses of MBI scores, however, resulted in higher performances: mean R^2^ = 0.388 (95% CI = 0.380 − 0.397), mean RMSE = 0.782 (95% CI = 0.777 − 0.787) in the training set, and mean R^2^ = 0.351 (95% CI = 0.332 − 0.370), mean RMSE = 0.805 (95% CI = 0.794 − 0.817) in the test set.

## Discussion

The aim of this study was to explore the factors associated with burnout in a sample of high school teachers and to build ML models that effectively predict the presence of burnout. Burnout was assessed using two questionnaires and, in the analysis, participants were assigned to the burnout group only if both instruments indicated the presence of burnout. Following this procedure, the prevalence of burnout in our sample of Hungarian high school teachers was 19.7%. In the literature, the prevalence of burnout in teachers is highly variable ranging between 9% and 53% [17, 64–66] but this range might even be wider as studies often do not report the prevalence of burnout [67]. In line with this, we also found an indication for a wider range, because the prevalence of burnout assessed by only MBI or MOLBI was also strongly different: 21.4% and 63%, respectively.

One of our main goals was to detect burnout using ML. The best predictive performance was achieved by the RF algorithm with class weights (AUC = 0.811), which is comparable with previous studies that aimed to predict burnout [34, 36, 39, 68]. Shavers et al., for example, who had also analyzed a teacher sample using a very similar approach reported an AUC score of 0.71 for the random forest algorithm on their test set [36]. Similarly, in another kind of sample in terms of occupation (i.e. physicians), Nishi et al. also reported an almost identical level of model performance with an AUC score of 0.72 [68]. In contrast, another study that involved healthcare workers reported a higher AUC score (i.e., 0.81), which is very similar to the best model performance in our research. Other studies with less specific samples reported relatively lower predictive performances of ML models predicting burnout [64]. The relatively good performance demonstrated by our models is probably due to the inclusion of psychological and psychiatric variables, the effects of which were less investigated by the mentioned previous studies.

Specifically, in our study various psychological and psychiatric variables had been included in the analyses and turned out to be important predictors of burnout. Especially depression and anxiety were consistently selected as highly important predictors, which is most probably due to the high comorbidities of burnout and the abovementioned psychiatric conditions [69–71]. The importance of these variables was additionally highlighted in the visualization of decision trees (see Fig. 2). In the presented decision trees, high levels of depression and anxiety symptoms were always associated with burnout, and they also tended to overshadow the importance of other factors that showed a smaller influence on the classification. However, it is important to note that we only presented two decision trees (i.e. the ones closest to the average model in terms of performance) out of the many decision trees created over the iterations and other trees might have had different structures. Regardless of this, the predictive values of depression, anxiety, and insomnia were remarkable. There was, however, another, more complex variable, CHS, which turned out to be one of the best predictors of burnout.

Feature selection indicated that CHS was among the most important variables used by the ML models. CHS reflects the respondents’ subjective evaluation of their current health status. Based on descriptive statistics and elastic net regression, higher levels of burnout were associated with lower levels of self-reported health status. This is in line with previous studies that found a negative relationship between self-reported health status and burnout [72, 73]. Thus, our result – consistent with the literature - highlights that burnt out teachers’ subjective evaluation of their own health status is relatively low. This is an especially important finding given the positive relationship between teachers’ self-reported health status and life satisfaction, which is especially strong in European countries (e.g. Hungary) [74] where the government spends relatively less on health care [75].

The subjectively evaluated status of current health is a complex indicator, which is affected by multiple factors [76]. Health-related complaints certainly play a crucial role in one’s subjective evaluation of their health status and in line with this, besides CHS, the EQ-5D subscales were also among the most important predictors of burnout. Descriptive statistics showed that complaints regarding mobility, pain\discomfort and usual activities were more enhanced in the burnout group (see Table 2), which gained further support in the regression analyses. This is in accordance with the literature because burnout is often characterized by sleep disturbances, low energy, and mental as well as physical fatigue [77–79] that have a profound negative impact on daily functioning [80]. In addition, several studies found that burnout was associated with physical complaints, for example, with stomach pain [79], headaches [81] and musculoskeletal pain [82].

Internet addiction-related variables such as the subscales of PIUQ (control, neglect, and obsession) and a related metric, the time spent online were all selected as important features as well. Based on descriptive statistics, mean values in all three aspects of internet addiction as well as the time spent online were higher in burnt out teachers, which was suggested by the results of regression as well. The association between internet addiction and burnout has been showed in both students [83, 84] and teachers [85]. In a Japanese sample of junior high school teachers, participants at risk of internet addiction had higher levels of depersonalization (i.e. an aspect of burnout) compared to those not at risk of internet addiction. In general, the association between addictive behaviour and burnout has been revealed in different contexts, for example, in alcoholism [10], social media addiction [86], or workaholism [87], however, in some cases, the strength of the association varies more strongly across studies, for example, in the case of substance use [88]. Although our research included only one cross-sectional study, the use of ML allowed us to get an insight into the variability of the strength of the association between internet addiction and burnout. The fact that control, neglect, and obsession as well as the time spent on the internet were all consistently among the best predictors regardless of which data analytic approach was used, suggests that the relationship between internet addiction and burnout is relatively strong.

Finally, work-related variables such as weekly working hours and the number of working years were also among the top predictors of burnout. The positive association between work hours and burnout symptoms is well known (see e.g. [89, 90]) and is in line with the basic characteristics of burnout. However, the relationship between burnout and working years is less straightforward according to the literature. In our research, regression analyses suggested that the longer one has been working as a teacher, the more severe their burnout symptoms are. Previous studies, however, found no association between burnout and teaching experience [91, 92] or found a rather non-linear trend suggesting that after increasing levels of burnout in the first decade of teaching are followed by a decreasing trend [93] In addition, we also found that age was negatively associated with burnout suggesting that particularly the time spent with teaching and not chronological age is a risk factor of burnout. This is in accordance with a similar study that investigated burnout in Hungarian teachers showing that age was negatively associated with disengagement, which is a central aspect of burnout [94].

### Limitations and future directions for research

Although this study provides findings that significantly contribute to a better understanding of high school teachers’ burnout, certain limitations need to be acknowledged. First, in this study, only self-report measures were used for both the calculation of the outcome variable and the predictors, which were therefore prone to the same kind of biases. In addition, the natural inaccuracies in self-reporting might have limited the predictive performance of the models. Therefore, in the future, the efficacy of the models perhaps could be improved by the inclusion of objective metrics, for example, physiological data such as heart rate variability [95] or electroencephalographic data [96]. Another limitation is that we only tested Hungarian high school teachers and thus, we could not estimate how well the results generalize to another population. Future studies might consider testing between-site generalizability by training the ML algorithms on data from one location and testing them on data collected in a different location, for example, in a different country. Using this methodological approach would potentially shed light on interesting cultural differences or consistencies.

## Conclusions

To conclude, despite the class imbalance (i.e. the natural underrepresentation of burnout in the sample), ML models were found to be able to effectively detect burnout in a sample of high school teachers. Notably, good model performances were found in the previously unseen holdout samples suggesting that the models could potentially be used in practice as well; however, follow-up studies are required to get a complete picture of the models’ generalizability. The most important predictors were variables related to psychiatric constructs such as depression, anxiety, insomnia and internet addiction as well as work-related variables such as how long one has been teaching and weekly working hours. In addition, other health-related variables that measure the subjective health status, mobility and everyday functioning of the participants were also predictive of burnout.

### Electronic supplementary material

Below is the link to the electronic supplementary material.

Supplementary Material 1

## Data Availability

The dataset analyzed in this study is available at the following site: https://data.mendeley.com/datasets/2yy4j7rgvg/2.
