# Supplementary material for: Mental issues, internet addiction and quality of life predict burnout among Hungarian teachers: a machine learning analysis
Source: BMC Public Health. 2024 Aug 27;24:2322. doi: 10.1186/s12889-024-19797-9 (PMC11348772; doi:10.1186/s12889-024-19797-9)
Supplement: Supplementary file 1 — Supplementary Material 1 [file 12889_2024_19797_MOESM1_ESM.docx]

**Supplementary materials**

**Mental issues, internet addiction and quality of life predict burnout among Hungarian teachers: a machine learning analysis**

Gergely Feher^1^, Krisztian Kapus^1^, Antal Tibold^1^, Zoltan Banko^2^, Gyula Berke^2^, Boroka Gacs^3^, Imre Varadi^1^, Rita Nyulas^4^, Andras Matuz^3,5 *^

^1^Centre for Occupational Medicine, Medical School, University of Pécs, Pécs, Hungary

^2^Department of Labour Law and Social Security Law, Faculty of Law, University of Pécs, Pécs, Hungary

^3^Department of Behavioural Sciences, Medical School, University of Pécs, Pécs, Hungary

^4^Baranya County SZC Zipernowsky Károly Technical College, Pécs, Hungary

^5^Szentágothai Research Centre, University of Pécs, Pécs, Hungary

^*^ Correspondence:

András Matuz, Ph.D.

Department of Behavioural Sciences, Medical School

University of Pécs, Szigeti str. 12, Pécs, 7624, Hungary

e-mail: [andras.matuz@aok.pte.hu](mailto:arpad.csatho@aok.pte.hu)

Phone: +(36) 72 536

**Table S1.** List of questions and response-options involved in the analysis.

| **Variable** | **Response options** |
| --- | --- |
| *Sociodemographic questions* |  |
| Gender | Categorical (2 categories) |
| Age | Categorical (6 categories) |
| Family status | Categorical (4 categories) |
| Number of children | Categorical (4 categories) |
| Education | Categorical (4 categories) |
| Work years | Categorical (7 categories) |
| Work schedule | Categorical (2 categories) |
| Working hours\week | Numerical |
| *Health-related questions \ Diagnoses* |  |
| Regular medication | Categorical (Yes/No) |
| Smoking | Categorical (Yes/No) |
| Alcohol consumption | Categorical (Yes/No) |
| Substance use | Categorical (Yes/No) |
| Diabetes | Categorical (Yes/No) |
| Hypertonia | Categorical (Yes/No) |
| Cardiac disease | Categorical (Yes/No) |
| Musculoskeletal disorder | Categorical (Yes/No) |
| Cancer | Categorical (Yes/No) |
| Mental disorder | Categorical (Yes/No) |
| Disabilities | Categorical (8 categories, multiple choice) |
| EuroQol5 Self-sufficiency | Likert-scale (5 points) |
| EuroQol5 Usual activities | Likert-scale (5 points) |
| EuroQol5 Mobility | Likert-scale (5 points) |
| EuroQol5 Anxiety\Depression | Likert-scale (5 points) |
| EuroQol5 Pain\Malaise | Likert-scale (5 points) |
| Current health status | Numerical |
| *Internet use related questions* |  |
| Daily time spent on the internet | Numerical |
| Time intervals online | Categorical (8 categories, multiple choice) |
| Goals of being online | Categorical (8 categories, multiple choice) |
| PIUQ Control disorder | Numerical |
| PIUQ Neglect | Numerical |
| PIUQ Obsession | Numerical |
| *Other psychiatric questionnaires* |  |
| BDI | Numerical |
| AIS | Numerical |

*Note: AIS = Athens Insomnia Scale sum score; BDI = Beck’s Depression Inventory sum score; PIUQ = Problematic Internet Use Questionnaire*

**Figure S1.** Feature selection via recursive feature elimination when burnout categories were assigned based on Maslach Burnout Inventory only.


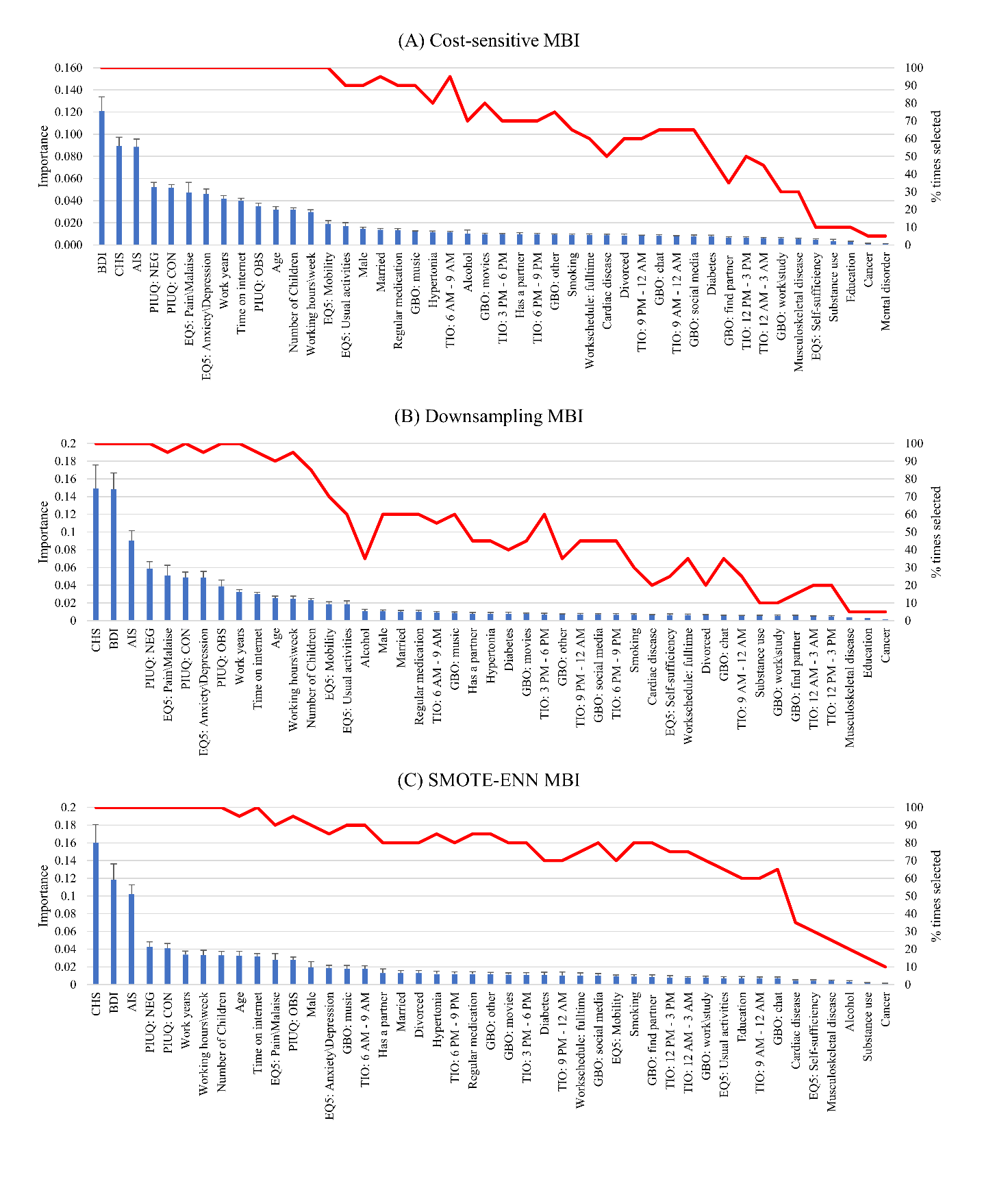


**Figure S2.** Feature selection via recursive feature elimination when burnout categories were assigned based on Mini Oldenburg Burnout Inventory only.


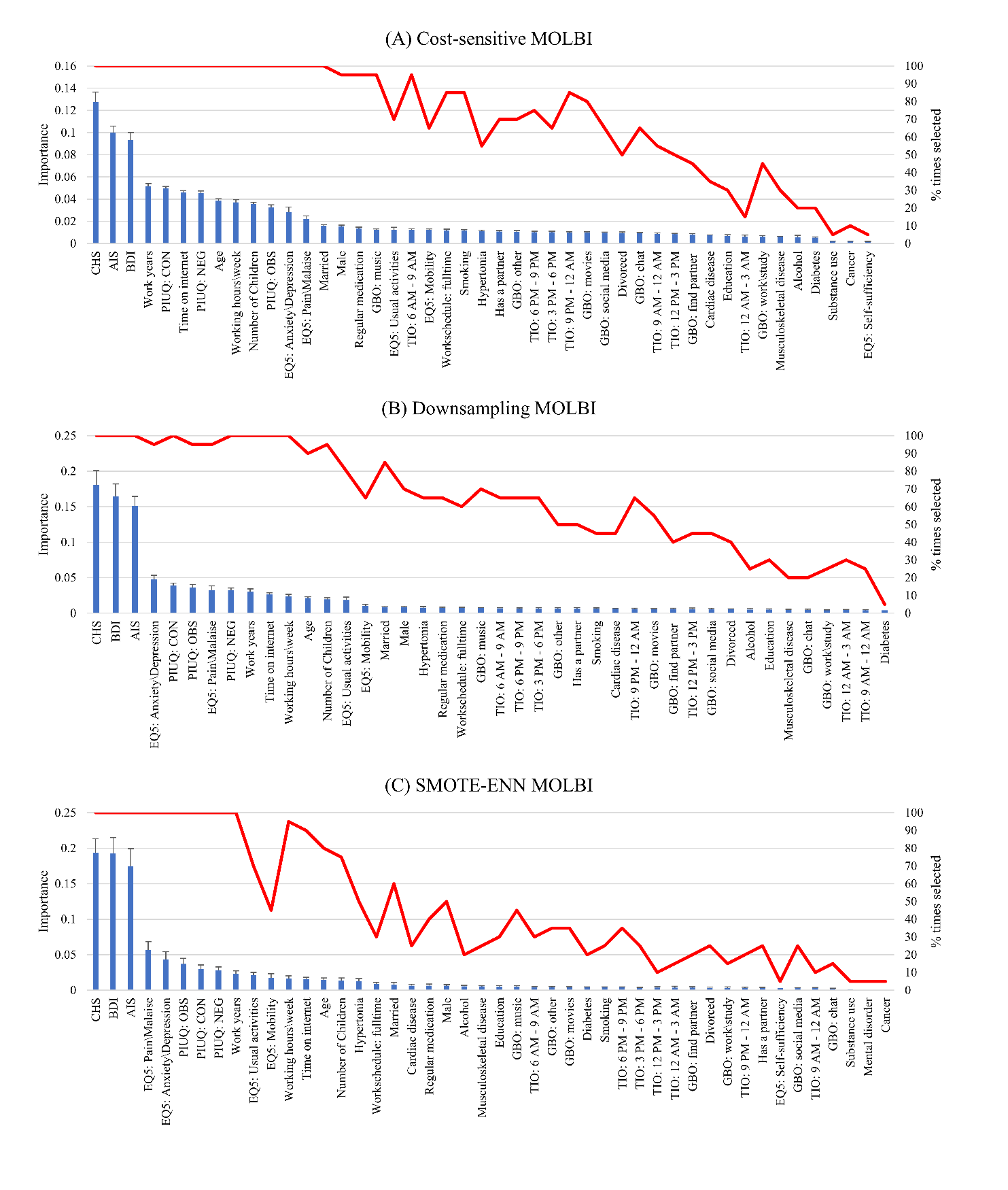


**Table S2**. Results of classification algorithms predicting Maslach Burnout Inventory based burnout

|  |  | Evaluation metrics | | | |
| --- | --- | --- | --- | --- | --- |
| ***Algorithm*** | ***Dataset*** | ***Balanced accuracy  (95% CI)*** | ***AUC  (95% CI)*** | ***Sensitivity  (95% CI)*** | ***Specificity  (95% CI)*** |
| *Cost-sensitive* | | | | | |
| Baseline model | Training | .726 (.719 - .733) | .819 (.813 - .825) | .616 (.602 - .630) | .836 (.833 - .839) |
|  | Test | .699 (.689 - .709) | .777 (.765 - .789) | .569 (.551 - .587) | .829 (.821 - .837) |
| Decision tree | Training | .754 (.747 - .761) | .804 (.798 - .810) | .739 (.704 - .774) | .770 (.744 - .796) |
|  | Test | .713 (.699 - .727) | .763 (.750 - .776) | .677 (.639 - .715) | .748 (.721 - .775) |
| Random forest | Training | .805 (.790 - .820) | .882 (.867 - .897) | .792 (.769 - .815) | .819 (.810 - .828) |
|  | Test | .720 (.705 - .735) | .795 (.783 - .807) | .674 (.648 - .700) | .767 (.754 - .780) |
| Support vector machine | Training | .738 (.705 - .771) | .868 (.853 - .883) | .576 (.486 - .666) | .901 (.872 - .930) |
|  | Test | .667 (.643 - .691) | .781 (.770 - .792) | .458 (.378 - .538) | .875 (.841 - .909) |
| *Downsampling* | | | | | |
| Baseline model | Training | .810 (.799 - .821) | .896 (.889 - .903) | .691 (.672 - .710) | .929 (.923 - .935) |
|  | Test | .705 (.694 - .716) | .768 (.754 - .782) | .650 (.629 - .671) | .759 (.742 - .776) |
| Decision tree | Training | .736 (.728 - .744) | .784 (.776 - .792) | .729 (.698 - .760) | .743 (.708 - .778) |
|  | Test | .695 (.679 - .711) | .739 (.723 - .755) | .662 (.621 - .703) | .728 (.689 - .767) |
| Random forest | Training | .757 (.747 - .767) | .834 (.825 - .843) | .716 (.687 - .745) | .797 (.779 - .815) |
|  | Test | .711 (.695 - .727) | .781 (.763 - .799) | .646 (.615 - .677) | .776 (.752 - .800) |
| Support vector machine | Training | .673 (.637 - .709) | .834 (.824 - .844) | .459 (.342 - .576) | .887 (.838 - .936) |
|  | Test | .640 (.610 - .670) | .773 (.760 - .786) | .400 (.294 - .506) | .880 (.829 - .931) |
| *SMOTE-ENN* | | | | | |
| Baseline model | Training | .831 (.824 - .838) | .913 (.907 - .919) | .884 (.874 - .894) | .779 (.769 - .789) |
|  | Test | .686 (.672 - .700) | .765 (.753 - .777) | .767 (.745 - .789) | .605 (.592 - .618) |
| Decision tree | Training | .717 (.708 - .726) | .774 (.763 - .785) | .790 (.775 - .805) | .645 (.619 - .671) |
|  | Test | .668 (.655 - .681) | .707 (.697 - .717) | .719 (.696 - .742) | .617 (.588 - .646) |
| Random forest | Training | .716 (.701 - .731) | .823 (.810 - .836) | .888 (.878 - .898) | .545 (.511 - .579) |
|  | Test | .670 (.661 - .679) | .756 (.745 - .767) | .829 (.800 - .858) | .510 (.477 - .543) |
| Support vector machine | Training | .720 (.695 - .745) | .809 (.801 - .817) | .848 (.823 - .873) | .591 (.522 - .66) |
|  | Test | .676 (.653 - .699) | .765 (.753 - .777) | .789 (.752 - .826) | .564 (.494 - .634) |

*Note: AUC = area under the receiver operating characteristic curve; CI = confidence interval; SMOTE-ENN = Synthetic Minority Over-sampling Technique with Edited Nearest Neighbours*

**Table S3**. Results of classification algorithms predicting Mini Oldenburg Burnout Inventory based burnout

|  |  | Evaluation metrics | | | |
| --- | --- | --- | --- | --- | --- |
| ***Algorithm*** | ***Dataset*** | ***Balanced accuracy  (95% CI)*** | ***AUC  (95% CI)*** | ***Sensitivity  (95% CI)*** | ***Specificity  (95% CI)*** |
| *Cost-sensitive* | | | | | |
| Baseline model | Training | .738 (.734 - .742) | .830 (.827 - .833) | .812 (.808 - .816) | .664 (.657 - .671) |
|  | Test | .713 (.703 - .723) | .806 (.799 - .813) | .794 (.785 - .803) | .631 (.616 - .646) |
| Decision tree | Training | .706 (.694 - .718) | .810 (.803 - .817) | .845 (.810 - .880) | .566 (.511 - .621) |
|  | Test | .681 (.666 - .696) | .774 (.763 - .785) | .815 (.786 - .844) | .547 (.491 - .603) |
| Random forest | Training | .792 (.779 - .805) | .900 (.890 - .910) | .872 (.863 - .881) | .711 (.689 - .733) |
|  | Test | .713 (.705 - .721) | .815 (.808 - .822) | .833 (.822 - .844) | .593 (.576 - .610) |
| Support vector machine | Training | .763 (.754 - .772) | .850 (.840 - .860) | .819 (.808 - .830) | .707 (.695 - .719) |
|  | Test | .718 (.710 - .726) | .807 (.800 - .814) | .782 (.774 - .790) | .654 (.641 - .667) |
| *Downsampling* | | | | | |
| Baseline model | Training | .876 (.870 - .882) | .948 (.944 - .952) | .821 (.810 - .832) | .931 (.926 - .936) |
|  | Test | .707 (.702 - .712) | .796 (.787 - .805) | .499 (.487 - .511) | .914 (.905 - .923) |
| Decision tree | Training | .728 (.721 - .735) | .795 (.786 - .804) | .547 (.527 - .567) | .909 (.893 - .925) |
|  | Test | .701 (.693 - .709) | .762 (.752 - .772) | .519 (.493 - .545) | .884 (.869 - .899) |
| Random forest | Training | .775 (.769 - .781) | .857 (.851 - .863) | .594 (.586 - .602) | .957 (.950 - .964) |
|  | Test | .734 (.726 - .742) | .809 (.802 - .816) | .563 (.549 - .577) | .905 (.890 - .920) |
| Support vector machine | Training | .725 (.720 - .730) | .828 (.824 - .832) | .501 (.488 - .514) | .948 (.941 - .955) |
|  | Test | .707 (.700 - .714) | .806 (.798 - .814) | .484 (.468 - .500) | .930 (.923 - .937) |
| *SMOTE-ENN* | | | | | |
| Baseline model | Training | .928 (.921 - .935) | .979 (.976 - .982) | .897 (.885 - .909) | .959 (.953 - .965) |
|  | Test | .722 (.713 - .731) | .797 (.786 - .808) | .582 (.563 - .601) | .863 (.845 - .881) |
| Decision tree | Training | .732 (.726 - .738) | .769 (.761 - .777) | .627 (.597 - .657) | .838 (.814 - .862) |
|  | Test | .711 (.703 - .719) | .746 (.735 - .757) | .600 (.576 - .624) | .823 (.794 - .852) |
| Random forest | Training | .761 (.757 - .765) | .832 (.827 - .837) | .655 (.644 - .666) | .867 (.858 - .876) |
|  | Test | .736 (.730 - .742) | .804 (.797 - .811) | .630 (.620 - .640) | .843 (.828 - .858) |
| Support vector machine | Training | .735 (.729 - .741) | .822 (.818 - .826) | .567 (.546 - .588) | .902 (.889 - .915) |
|  | Test | .721 (.714 - .728) | .805 (.796 - .814) | .553 (.528 - .578) | .890 (.874 - .906) |

*Note: AUC = area under the receiver operating characteristic curve; CI = confidence interval; SMOTE-ENN = Synthetic Minority Over-sampling Technique with Edited Nearest Neighbours*
